# Identification of Chicken Bone Paste in Starch-Based Sausages Using Laser-Induced Breakdown Spectroscopy

**DOI:** 10.3390/s25134226

**Published:** 2025-07-07

**Authors:** Haoyu Li, Li Shen, Xiang Han, Yu Liu, Yutong Wang

**Affiliations:** Tianjin Key Laboratory of Quantum Optics and Intelligent Photonics, School of Materials Science and Engineering, Tianjin University of Technology, Tianjin 300384, China; lihaoyu934@126.com (H.L.); hanxiangtut@163.com (X.H.); liuyutut@163.com (Y.L.); hiadina@163.com (Y.W.)

**Keywords:** laser-induced breakdown spectroscopy, starch sausage, chicken bone paste, food identification

## Abstract

This study aims to rapidly in situ identify starch sausage samples with the improper addition of chicken bone paste. Chicken bones play important roles in building materials, biomass fuels, and as food additives after enzymatic hydrolysis, but no current research indicates that chicken bones can be directly added to food for consumption. Especially in starch sausages, the addition of chicken bone paste is highly controversial due to potential risks of esophageal laceration and religious concerns. This paper first uses laser-induced breakdown spectroscopy (LIBS) to investigate the elemental differences between starch sausages and chicken bone paste. By preparing mixtures of starch sausages and chicken bone paste at different ratios, the relationships between the spectral peak intensities of elements, such as Ca, Ba, and Sr, and the proportion of chicken bone paste were determined. Through processing methods such as normalization with reference spectral lines, selection of the signal of the second laser pulse at the same position, and electron temperature correction, the determination coefficients (R^2^) of each element’s spectral lines have significantly improved. Specifically, the R^2^ values for Ca I, Ca II, Ba II, and Sr II have increased from 0.302, 0.694, 0.857, and 0.691 to 0.972, 0.952, 0.970, and 0.982, respectively. Finally, principal component analysis (PCA) was used to distinguish starch sausages, chicken bone paste, and their mixtures at different ratios, with further effective differentiation achieved through t-distributed stochastic neighbor embedding (t-SNE). The results show that LIBS technology can serve as an effective and rapid method for detecting elemental composition in food and distinguishing different food products, providing safety guarantees for food production and supervision.

## 1. Introduction

Chicken is widely consumed for its rich nutritional value and relatively lower cost compared to other meats. Analyses show that chicken has significant impacts on the economy [1], health [2], culture [3], and religious beliefs [4]. With the extensive use of chicken, the proper disposal of chicken bones to enhance their utilization value has become a key issue. Chicken bones play important roles in extracting collagen for gelatin production [5], reducing oil absorption in fried chicken [6], and extracting peptide-calcium chelates [7]. However, chicken bones generally cannot be directly added to food, as this may lead to bacterial contamination [8], gastrointestinal foreign bodies [9], the generation of a large amount of animal-derived waste [10,11], and religious issues [4]. Furthermore, the ingestion of bones by humans or pets can pose serious health and safety risks [12,13], especially the controversial practice of directly adding chicken bone paste to chicken starch sausages. Therefore, the need for an effective and rapid method to detect the improper addition of chicken bone paste in food is crucial.

In the field of meat adulteration detection, there are many different types of analytical methods. Among them, polymerase chain reaction (PCR) [14] and loop-mediated isothermal amplification (LAMP) [15] are the most reliable and widely used DNA- and protein-based detection techniques. In recent years, spectroscopic methods such as mid-infrared spectroscopy [16], near-infrared reflectance spectroscopy [17], and Raman spectroscopy [18] have also gradually gained extensive application in meat identification. These spectroscopic techniques primarily rely on differences in molecular structure or functional groups to identify meats. However, they exhibit significant limitations in effectively detecting elemental composition in meat products, especially excessive heavy metals. Although DNA- and protein-based detection methods are the most commonly used for identifying meat adulteration and offer advantages such as high sensitivity and reliability, they are often associated with issues like high cost, long detection time, and complicated sample preparation processes. Therefore, there is an urgent need for a detection method that is not only rapid but also accurate and sensitive to support research on meat adulteration.

In recent years, laser-induced breakdown spectroscopy (LIBS), a technology based on the emission of light from atoms, ions, and molecular radicals, has been widely applied in Mars exploration [19], deep-sea detection [20], and pharmaceutical analysis [21] due to its advantages of rapid analysis, in situ measurement, and no need for sample preparation. Especially in the field of food analysis, LIBS has been used for qualitative and quantitative analysis of milk [22], food color analysis [23], applications in mineral identification and automated mineral recognition [24], and the highly sensitive detection of lead chromate green and lead in tea [25]. Notably, principal component analysis (PCA) and partial least squares (PLS) can enhance LIBS analysis performance and provide more valuable information. For meat screening, an increasing number of studies have combined LIBS with these methods. For example, Bilge et al. used LIBS combined with PCA, PLS, and independent analogy models for the qualitative identification of meat types, achieving an identification rate of 83.37% [26]. Velioglu et al. used LIBS to distinguish pure beef from offal samples, successfully differentiating beef offal and mixed offal via PCA and determining adulteration ratios using PLS with a coefficient of determination (R^2^) of 0.947 and a limit of detection (LoD) of 3.8% [27]. Sezer et al. established LIBS spectra based on proteins to identify beef, chicken, and pork, evaluating clustering patterns of meats using PCA, determining adulteration ratios with PLS, and calculating LoDs for chicken and pork adulteration in beef as 2.84% and 3.89%, respectively, via the bulk protein method [28]. However, there has been no reported use of LIBS technology for detecting the improper addition of chicken bone paste in chicken starch sausages. Additionally, as a nonlinear dimensionality reduction method for high-dimensional data visualization, t-distributed stochastic neighbor embedding (t-SNE) outperforms methods such as PCA, independent component analysis (ICA), and local semantic analysis (LSA) in classification effects. For example, Yao et al. combined LIBS with t-SNE and a backpropagation neural network (BPNN) to classify and trace compound cold medicines containing paracetamol and amantadine hydrochloride with the same principal components, achieving an identification accuracy of 96.7% [29].

Based on the above analysis, this paper aims to rapidly, in situ, and accurately identify chicken starch sausage samples with the improper addition of chicken bone paste. First, elemental differences between chicken starch sausages and chicken bone paste were analyzed through their LIBS spectra. Second, by examining the relationships between the spectral peak intensities of elements such as Ca, Ba, and Sr and the ratio of chicken starch sausage to chicken bone paste, the main characteristic elements in chicken bone paste were further determined. Finally, PCA and t-SNE were used to achieve effective differentiation among chicken starch sausages, chicken bone paste, and their mixtures at different ratios.

## 2. Materials and Methods

### 2.1. Experimental Setup

**Figure 1:**
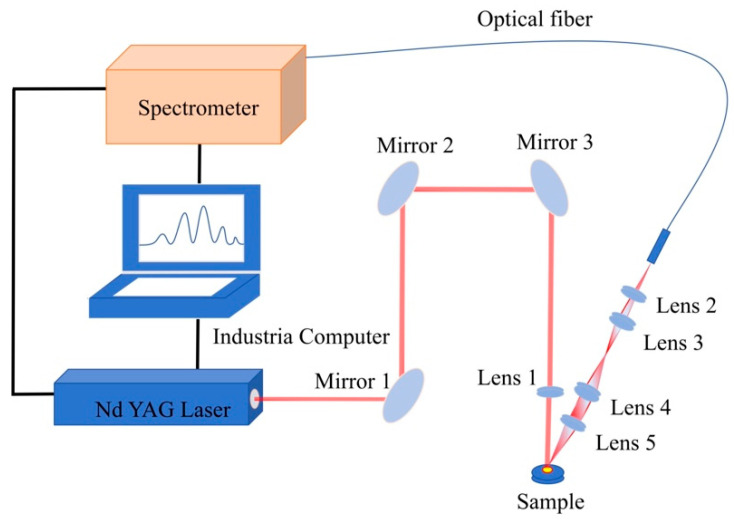
Schematic diagram of the LIBS experimental setup.

The configuration of our LIBS experimental setup was designed by referencing the previous works conducted in our laboratory [25,29]. The LIBS experimental setup is shown in Figure 1. A pulsed signal generated by a computer triggers a homemade Nd:YAG laser to produce laser pulses with a wavelength of 1064 nm, pulse energy of approximately 100 mJ, and pulse width of approximately 20 ns. The voltage of the laser flashlamp was 700 V, and the relative standard deviation (RSD) of the energy of 50 laser pulses was 2.89%. After passing through three mirrors, the pulsed laser is focused by a plano-convex lens with a focal length of 60 mm onto the surface of starch sausage–chicken bone paste mixed samples placed on a sample stage, with the distance between the lens and the sample surface set at 58 mm. The high-energy pulsed laser ablates the sample surface to generate plasma, creating ablation craters smaller than 100 μm and an irradiance exceeding 6.3 × 10^10^ W·cm^−2^. Four plano-convex lenses with different focal lengths couple the emitted light from plasma de-excitation into an optical fiber, which is then transmitted to a four-channel CCD spectrometer (AvaSpec-2048-USB2*). The spectrometer is triggered by the pulsed signal from the laser power supply. The spectral range collected by the spectrometer is 200–950 nm, with a spectral resolution of 0.10–0.15 nm across five channels. To improve the fiber-coupling efficiency and collect more plasma radiation spectra, the focal lengths of the four plano-convex lenses in the light collection system are 60 mm, 150 mm, 60 mm, and 10 mm, respectively. To reduce the influence of bremsstrahlung and recombination radiation on LIBS spectra, the spectrometer starts collecting optical signals 1.3 μs after the laser interacts with the sample, with an integration time set to 1.05 ms [25,29]. Tablets containing starch sausage–chicken bone paste mixtures are fixed on a two-dimensional electrically controlled displacement stage using transparent tape. To avoid interference between different ablation craters, the interval between craters is set to 0.5 mm. To minimize the impact of laser energy fluctuations and sample surface flatness on the signals, 121 samples are taken from different parts of the starch sausage-chicken bone paste tablets, with only one pulse applied to the same position. The final averaged spectrum is obtained through signal processing.

### 2.2. Sample Preparation

The main protein component of starch sausages is mostly chicken, which contains low fat, cholesterol, and sodium. Starch sausage is a product developed and marketed by Henan Shuanghui Investment & Development Co. Ltd. (Luohe, Henan Province, China) and the chicken bone paste was obtained from commercially available poultry raw materials. The starch sausages were sliced and ground into powder using a wall-breaking machine. Due to the high moisture and oil content, the chicken bone paste was heated on a heating plate to 80 °C and maintained for 40 min, then ground into powder in a wall-breaking machine. The starch sausage powder and chicken bone paste powder were mixed at mass ratios of 9:1, 8:2, 7:3, 6:4, and 5:5 to form 1 g samples each. These mixtures were pressed into uniform circular tablets with a diameter of 10 mm using a tablet press at a pressure of 5 MPa for 30 s. Additionally, 1 g of pure starch sausage powder and chicken bone paste powder were separately pressed into the same type of tablets as reference samples.

### 2.3. Methods

#### 2.3.1. PCA Analysis

LIBS spectra are large and multidimensional datasets, and the LIBS spectrum of each sample or class is represented by unique spectral characteristics and multiple element information, the so-called “chemical fingerprints” [30]. PCA is a well-established method for the multidimensional classification of complex and large datasets, which is often used for LIBS data classification [31]. PCA is an unsupervised statistical analysis technique that finds linear combinations of variables, i.e., principal components (PC), which describe major trends in the dataset. PCA provides a useful tool for identifying whether samples are the same or different, and which variables are responsible for observed differences, but is not generally appropriate for classification of unknown data [32]. In this paper, PCA was used to identify and classify pure starch sausage and chicken bone paste samples.

#### 2.3.2. t-SNE Analysis

The t-SNE was applied to the dimensionality reduction analysis of these starch sausage and chicken bone paste samples. As a method of dimensionality reduction in high-dimensional data, it is a probability-based method for evaluating the similarity between high-dimensional data points and utilizing low-dimensional spaces to maintain that similarity [33]. Meanwhile, t-SNE solves the problem of crowded sample distribution and indistinct boundaries in low-dimensional space, and t-SNE not only preserves the local structure of the data but also reacts to some key global structures. In the analysis process, perplexity is a parameter that measures the effective number of neighbors in a high-dimensional space. The greater the perplexity, the greater the local number of neighbors it involves.

#### 2.3.3. Electron Temperature Analysis

Laser-induced plasma has three important parameters: plasma density, electron temperature, and electron density. Under the assumptions of stoichiometric ablation, local thermal equilibrium, and optically thin conditions, the relationship between the integrated intensity *I_ji_* of elemental spectral lines in the plasma and the upper energy level *E_j_* and lower energy level *E_i_* of the element can be expressed as [34]:

(1) $$I_{ji}=A_{ji}n_{j}^{s}=n^{s}A_{ji}g_{j}\frac{e^{-\frac{E_{j}}{kT}}}{U_{s}(T)},$$

where $n_{j}^{s}$ is the number density of the element at energy level *E_j_*, *n^S^* is the total number density of the element, *A_ji_* is the transition probability, *g_j_* is the upper energy level degeneracy, *k* is the Boltzmann constant, *T* is the electron temperature, and *U_S_*(*T*) is the partition function of the element at electron temperature *T*, which can be calculated from the electron temperature through the NIST website.

According to the Saha equation, when atomic lines and ionic lines coexist, the ratio of ionic density *n^II^* to atomic density *n^I^* is:

(2) $$\frac{n^{II}}{n^{I}}=\frac{(2\pi m_{e}kT{)}^{3/2}}{n_{e}h^{3}}\frac{2U^{II}(T)}{U^{I}(T)}e^{-\frac{E_{ion}}{kT}},$$

where *n_e_* is the electron density, *E*_ion_ is the ionization energy of the atom, *m_e_* is the electron mass, *h* is Planck’s constant, and *U^I^*(*T*) and *U^II^*(*T*) are the partition functions of the atom and ion, respectively.

Thus, it can be seen that there is a relationship between atomic density, ion density, electron density, and electron temperature. Additionally, LIBS spectra in air generally exhibit H spectral lines. Due to the Stark effect, the linewidth of the H_α_ spectral line is related to electron density. The characteristics of Balmer lines and the low abundance of H ensure minimal self-absorption effects, and the Stark broadening of H spectral lines is an order of magnitude higher than that of heavy elements, significantly reducing measurement errors. The electron number density *n_e_* is [35]:

(3) $$n_{e}=8.02\times 10^{12}{\left(\frac{\Delta \lambda_{1/2}}{\alpha_{1/2}}\right)}^{3/2},$$

where Δ*λ*_1/2_ is the full width at half maximum (FWHM) of the spectral line, and *α*_1/2_ represents the FWHM of the simplified Stark line shape, which can be found in Table 1 of reference [36] or Table AIII.b of reference [37]. Therefore, when obtaining electron temperature through the Saha–Boltzmann plot, it is indeed necessary to consider the spectral linewidth.

It can be seen that the integrated intensity of spectral lines is related to electron temperature and element content. Therefore, when analyzing the relationship between the spectral line intensity of elements in mixed samples of different ratios and the doping ratio, the influence of electron temperature must be considered. Thus, we need to correct for the impact of electron temperature differences generated by different samples.

## 3. Results and Discussion

### 3.1. LIBS Spectral Analysis

**Figure 2:**
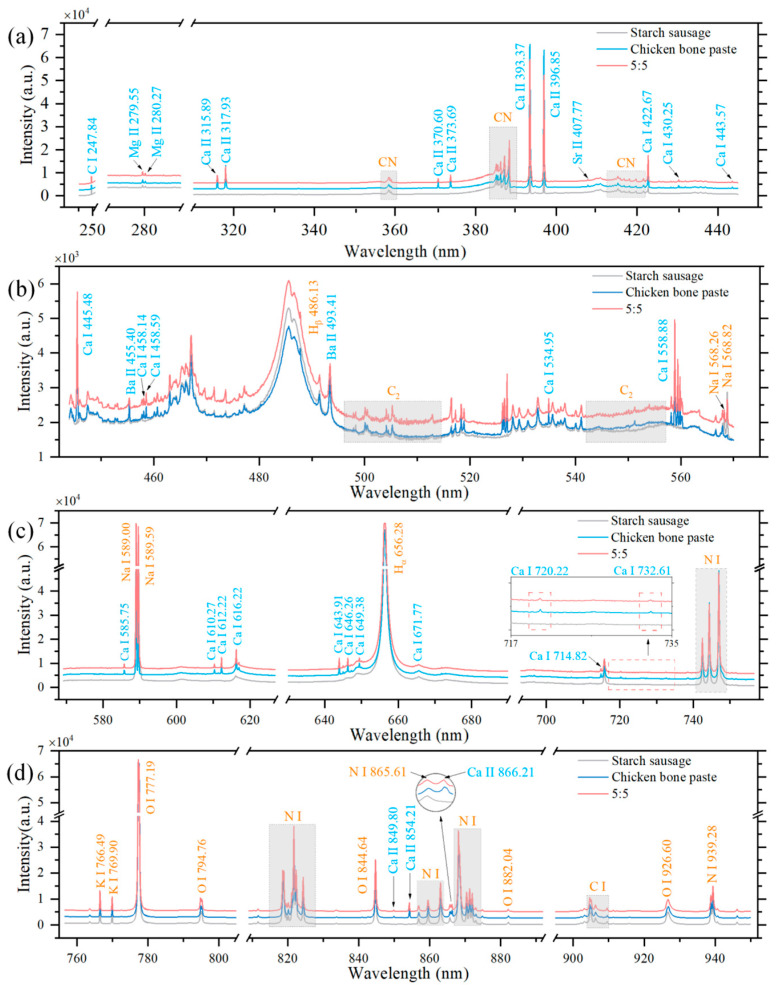
Averaged LIBS spectra of starch sausage, chicken bone paste, and the 5:5 ratio mixed sample under the second laser pulse. (**a**) 200−450 nm; (**b**) 442−575 nm; (**c**) 568−758 nm; and (**d**) 755−952 nm.

**Figure A1:**
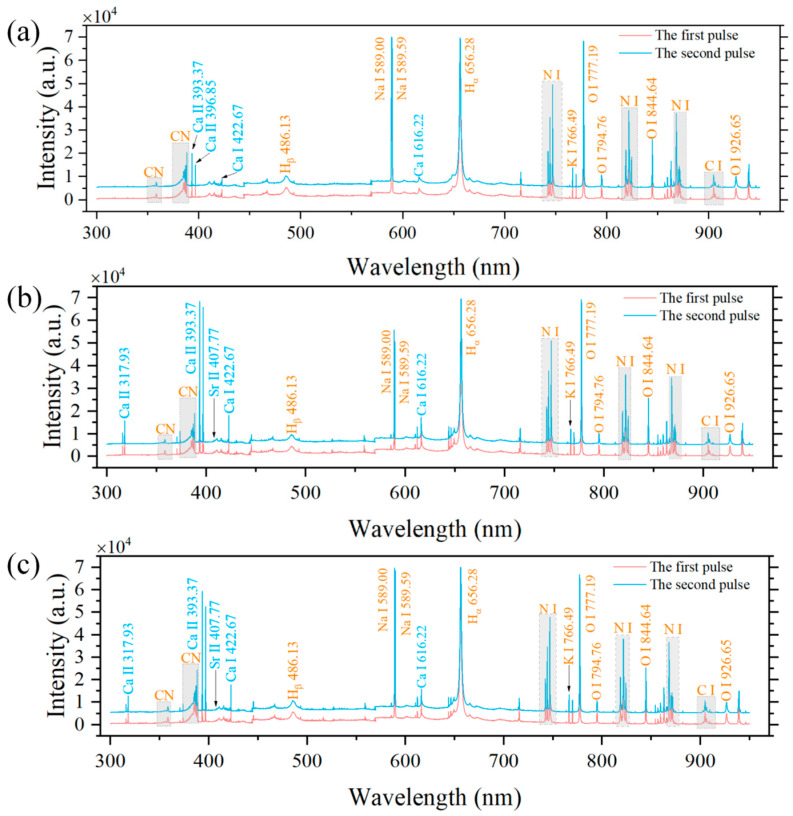
Averaged LIBS spectra under the first and second laser pulses. (**a**) Starch sausage; (**b**) chicken bone paste; and (**c**) the 5:5 ratio mixed sample.

The purpose of this study was to use LIBS technology to analyze the compositional differences between starch sausage and chicken bone paste samples and further accurately identify the improper addition of chicken bone paste in starch sausages. To this end, LIBS spectra of starch sausage, chicken bone paste, and a 5:5 mixed sample were first studied, with comparative analysis of spectral peak intensities of different elements. It is worth noting that due to potential surface contamination of samples during preparation and storage, which may affect the analysis results, this paper used LIBS spectra under the second laser pulse at the same sample position [38,39]. Figure A1 in Appendix A shows the LIBS spectra of the three samples under two laser pulses. Additionally, to minimize the impact of laser energy fluctuations and surface compositional uniformity on the results, 121 spectra obtained from different positions on the sample surface were averaged. Figure 2 displays the averaged LIBS spectra of starch sausage, chicken bone paste, and the 5:5 mixed sample under the second laser pulse. The National Institute of Standards and Technology (NIST) database was used to identify possible elemental or radical spectral lines in the LIBS spectra of different samples, with some stronger emission lines determined and listed in Table 1.

From Figure 2, the LIBS spectra show that macro elements such as K, Ca, and Na, trace elements such as Ba and Sr and compositional elements of organic compounds such as C, H, O, and N are present in both chicken bone paste and starch sausage samples. Additionally, due to the high content of organic components in chicken bone paste and starch sausages, spectral lines of CN and C_2_ radicals were observed in the LIBS spectra. The LIBS spectrum of the 5:5 ratio mixed sample exhibited spectral characteristics of both starch sausage and chicken bone paste. Notably, significant differences in the intensities of some elemental spectral peaks were observed between the two samples. For example, the spectral peak intensities of Ca atoms and ions in chicken bone paste were significantly stronger than those in starch sausage, primarily due to the higher Ca content in bones. Similarly, other alkaline earth metal elements such as Ba and Sr showed the same trend. However, for the alkali metals Na and K, the spectral peak signals were stronger in starch sausage.

### 3.2. Variation of Spectral Peak Intensities of Different Elements

**Figure 3:**
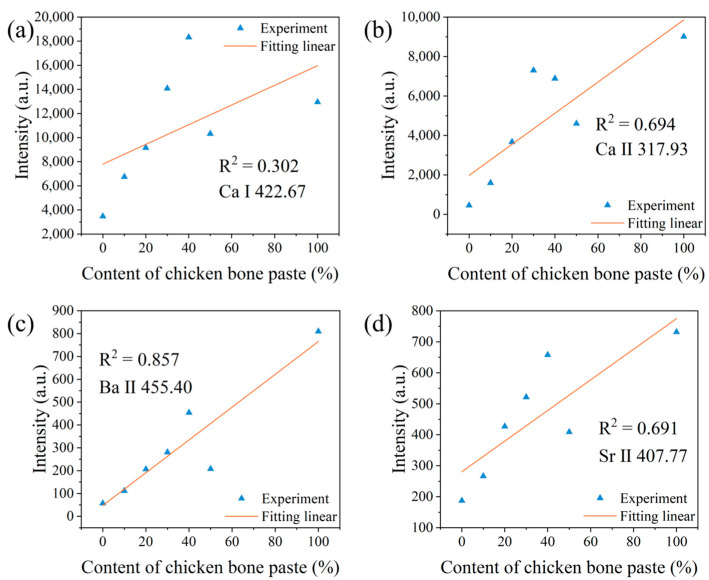
Relationships between the spectral peak intensities and the chicken bone paste content for the first laser pulse. (**a**) Ca I; (**b**) Ca II; (**c**) Ba II; and (**d**) Sr II.

To further verify the correlation between the spectral peak intensities of different elements and the samples, we analyzed the relationship between the spectral peak intensities of elements and the content of chicken bone paste. Figure 3 shows the relationship between the spectral peak intensities of Ca I, Ca II, Ba II, and Sr II and the content of chicken bone paste for the first laser pulse.

It can be seen that although the general rule is that the spectral peak intensities of these alkali metal elements increase as the content of chicken bone paste increases, the linear fitting effect is poor. The Ba II 455.40 nm spectral peak has the best fitting effect, with a coefficient of determination R^2^ of only 0.857, while the Ca I 422.67 nm spectral peak has the worst fitting effect, with a coefficient of determination R^2^ of only 0.302. Problems such as laser intensity fluctuations, sample surface flatness, and uniformity can cause fluctuations in the spectral signals during each measurement, resulting in an unsatisfactory fitting effect. Since the content of organic matter in starch sausages and chicken bone paste is relatively high, we selected the spectral peak of C I 247.86 nm, which is the main element in organic matter, as the reference peak to normalize the LIBS spectra. This can effectively suppress the influence of laser intensity fluctuations, sample surface flatness, and uniformity on the spectral signal fluctuations.

**Figure 4:**
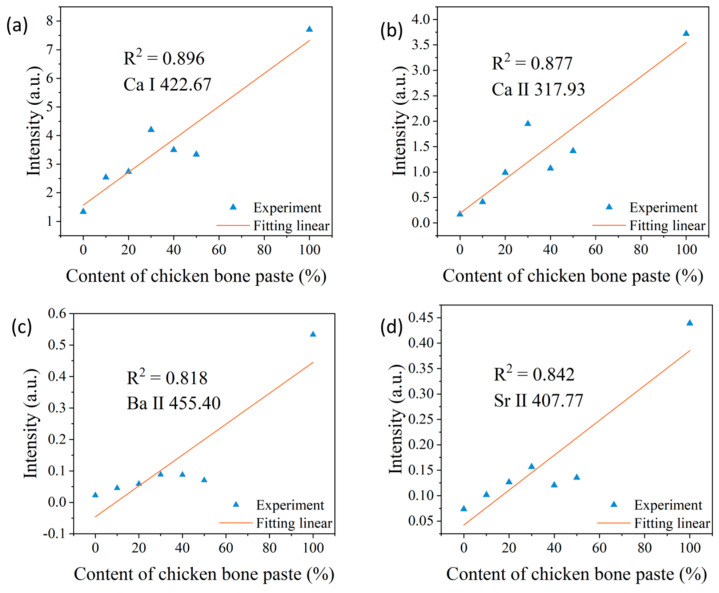
Relationships between the normalized spectral peak intensities and the chicken bone paste content for the first laser pulse. (**a**) Ca I; (**b**) Ca II; (**c**) Ba II; and (**d**) Sr II.

Figure 4 shows the relationships between the normalized spectral peak intensities of Ca I, Ca II, Ba II, and Sr II and the chicken bone paste content for the first laser pulse. It is evident that, compared with the non-normalized situation, after normalizing the LIBS spectra using the C I 247.86 nm spectral peak, the relationships between the spectral peak intensities of Ca I, Ca II, and Sr II and the chicken bone paste content, as well as the linear fitting effects, are significantly improved. For example, the coefficient of determination R^2^ of the linear fitting for the Ca I 422.67 nm spectral peak increases from 0.302 (non-normalized) to 0.896 (normalized). The linear fitting effect of the Ba II 455.40 nm spectral peak shows a slight decline, which may be due to the superposition of this spectral line on a relatively broad background signal and the fluctuations of the background signal. It is worth noting that surface contamination may occur during the sample preparation and storage processes, which can affect the analysis results. Therefore, at the same position, the first laser pulse is used to remove surface contaminants, and then the LIBS signal generated by the second laser pulse is used for analysis, which can eliminate the influence of surface contaminants on the results.

**Figure 5:**
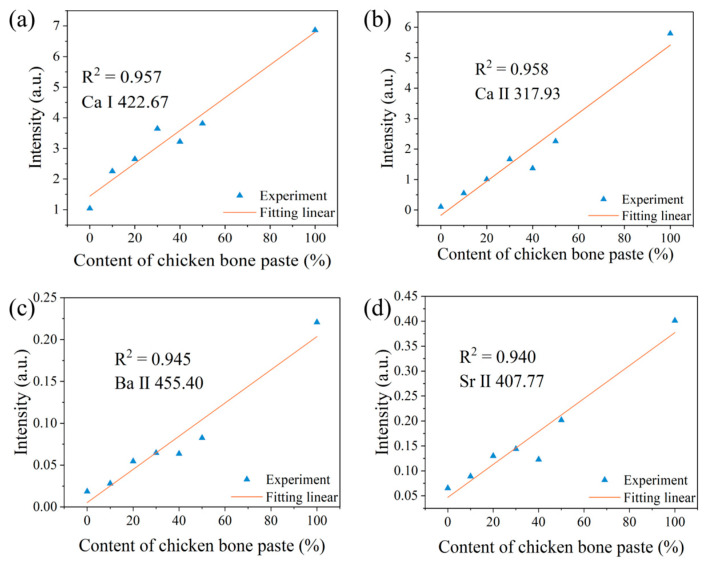
Relationships between the normalized spectral peak intensities and the chicken bone paste content for the second laser pulse. (**a**) Ca I; (**b**) Ca II; (**c**) Ba II; and (**d**) Sr II.

Figure 5 presents the relationships between the normalized spectral peak intensities of Ca I, Ca II, Ba II, and Sr II and the chicken bone paste content, along with linear fitting plots, during the second laser pulse. It can be seen that the coefficients of determination R^2^ are significantly improved for all elements. The Ca I 422.67 nm spectral peak exhibits the highest R^2^ value of 0.957 in linear fitting. This phenomenon may be attributed to two possible reasons: (1) this spectral peak is superimposed on a broad background spanning 230–300 nm (with a width of 70 nm), and the C I 247.86 nm spectral peak lies at the tail of this background, potentially affecting the normalized analysis results, and (2) the intensity of this spectral peak is weaker compared to the other four spectral peaks.

### 3.3. Correction with Electron Temperature

**Figure A2:**
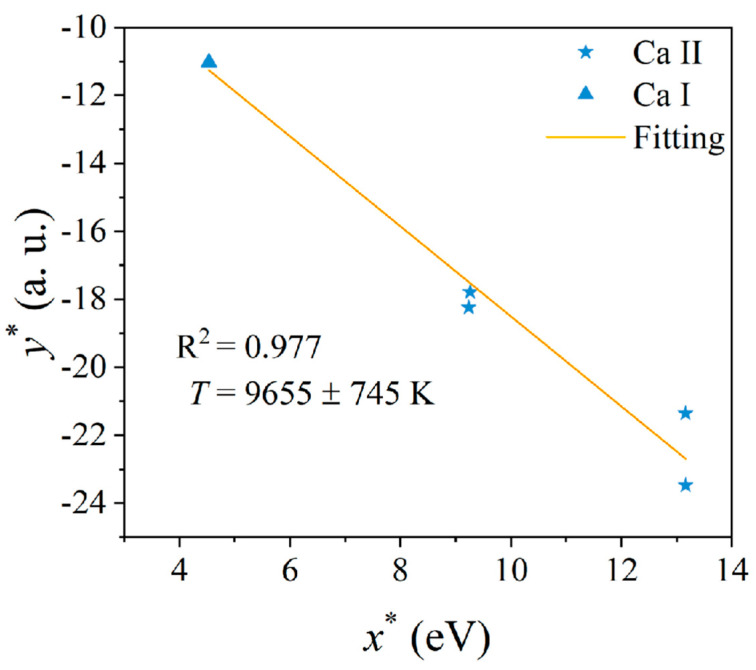
Saha−Boltzmann plots for the Ca in starch sausage sample.

The electron temperature can be obtained by plotting a Saha–Boltzmann plot. As analyzed, the Saha–Boltzmann plots for the Ca I and Ca II spectral lines in the starch sausage sample are shown in Figure A2. Through fitting, the obtained electron temperature is 9655 K. The electron temperatures fitted for all seven samples are listed in Table A1. It can be seen that except for the 4:6 sample, which has an electron temperature of 8759 K (below 9000 K), the electron temperatures of the other samples are relatively close, all greater than 9400 K. It is precisely due to these differences in electron temperature among the samples that the spectral peak intensity offsets are relatively large, particularly for the 4:6 sample.

**Figure 6:**
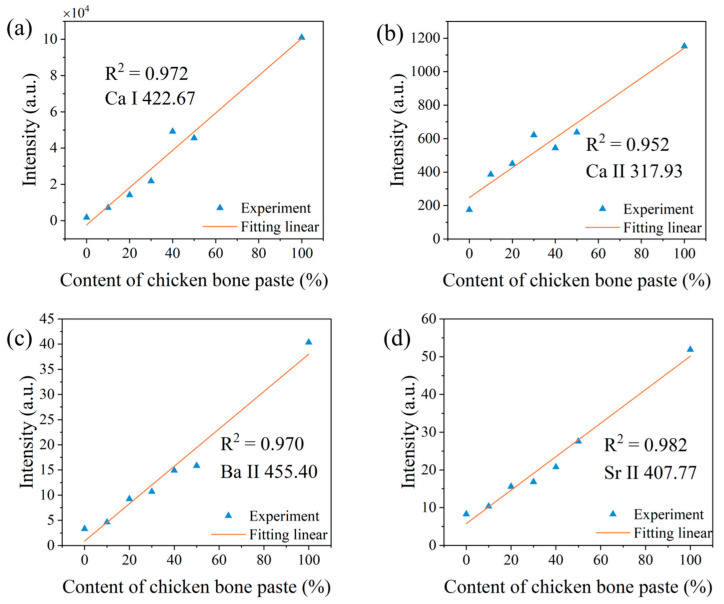
Relationships between the normalized spectral peak intensities and the chicken bone paste content after correction with electron temperature. (**a**) Ca I; (**b**) Ca II; (**c**) Ba II; and (**d**) Sr II.

Therefore, we need to correct for the influence of electron temperature on spectral peak intensity. According to Equation (1), the partition function *U_S_*(*T*) and the particle number density distribution function of the upper energy level *E_j_* were calculated. The corrected relationship between spectral peak intensity and sample ratios is shown in Figure 6. It can be seen that, except for the determination coefficient of Ca II 317.93 nm slightly decreasing from 0.958 to 0.952, the fitting determination coefficients of other spectral peak intensities have significantly improved, with specific values listed in Table 2. As shown in Figure 6, the deviation of the 4:6 sample from the fitted line has been greatly reduced, which confirms that the significant difference in electron temperature of this sample compared to others caused a large deviation between the spectral peak intensity of the analyzed elements and the fitted line. After correction, this deviation was significantly minimized, as shown in Figure 6. After considering this influence, the determination coefficients of all fitted lines are greater than 0.95, and the fitting results have been significantly improved.

### 3.4. Cluster Analysis

**Figure 7:**
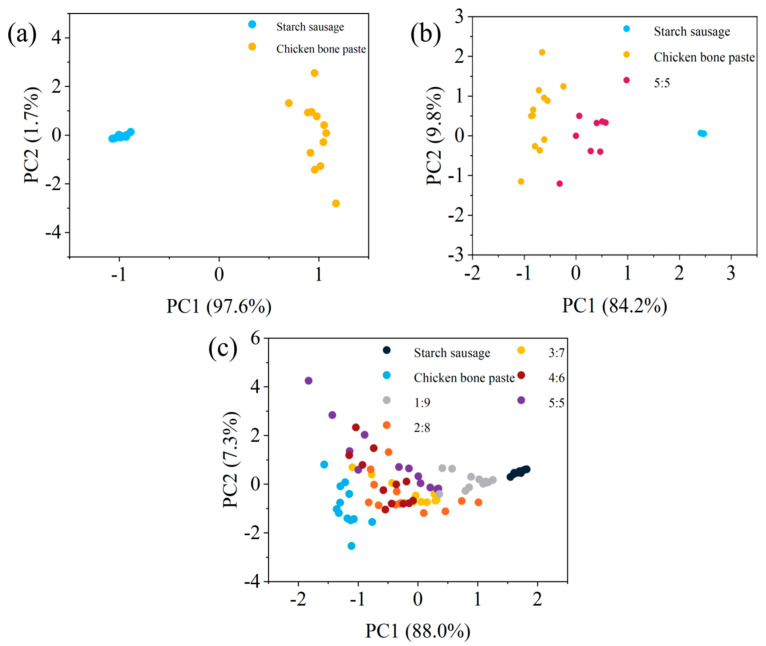
PCA analysis. (**a**) Starch sausage and chicken bone paste; (**b**) starch sausage, chicken bone paste, and 5:5 ratio mixed sample; (**c**) starch sausage, chicken bone paste, and five mixed samples.

In this paper, PCA was applied to perform cluster analysis on the LIBS spectra of starch sausages, chicken bone paste, and five mixed samples with different ratios. For each sample, 121 LIBS spectra under the second laser pulse (excluding the last spectrum) were averaged in groups of 10, resulting in 12 averaged spectra per sample. A total of 84 averaged spectra from the seven samples were subjected to PCA analysis, with the results shown in Figure 7. As seen in Figure 7a, when analyzing only starch sausage and chicken bone paste samples, the two categories can be clearly distinguished. When including the 5:5 mixed sample, Figure 7b shows that the three samples can still be differentiated. However, when performing PCA on all seven samples, Figure 7c reveals a poor clustering effect: only starch sausage and chicken bone paste samples are well-separated, while the other five mixed samples largely overlap and are difficult to distinguish effectively.

**Figure 8:**
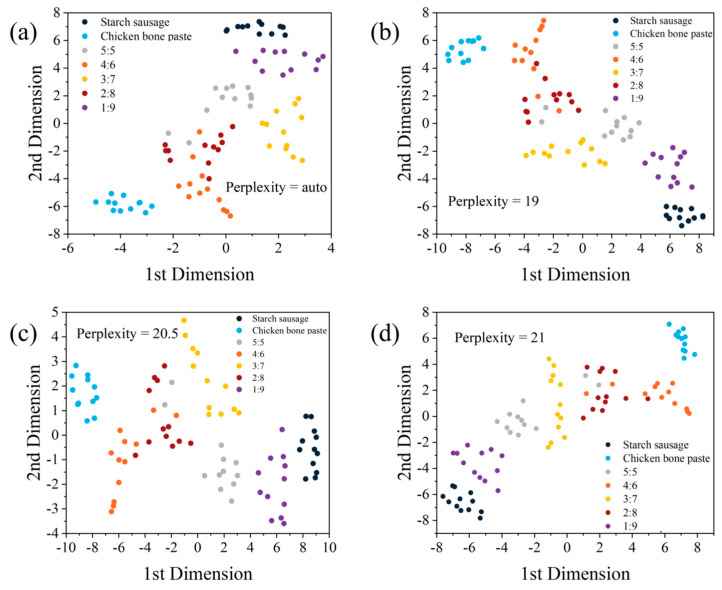
t-SNE analysis. (**a**) Perplexity = auto; (**b**) perplexity = 19; (**c**) perplexity = 20.5; (**d**) perplexity = 21.

Therefore, a more effective method is needed for dimensionality reduction analysis of starch sausage-chicken bone paste mixed samples with different ratios. As a nonlinear dimensionality reduction method for high-dimensional data, t-SNE is a probability-based approach used to evaluate the similarity between high-dimensional data points and preserve this similarity in a low-dimensional space [40]. Figure 8 shows the t-SNE analysis results for the seven samples. It can be seen that the clustering effect is significantly improved compared to PCA: when selecting different perplexity values, the overlap between data points is minimal, basically achieving effective differentiation among these samples.

## 4. Conclusions

To achieve in situ and rapid identification of starch sausage samples with improper addition of chicken bone paste, this study used LIBS to analyze starch sausage, chicken bone paste, and mixed samples with different ratios. First, through LIBS spectral analysis, spectral differences were found between starch sausage and chicken bone paste in alkaline earth metal elements (Ca, Ba, and Sr) and alkali metal elements (Na and K). Second, to minimize the effects of laser energy fluctuations, sample surface flatness and homogeneity, and potential contaminants, the spectral peaks under the second laser pulse at the same position were selected, and the C I 247.86 nm spectral peak was used as a reference peak for normalization. This obtained the normalized relationships between the spectral peak intensities of Ca, Ba, and Sr and the chicken bone paste content. Finally, clustering algorithms such as PCA and t-SNE were used to achieve effective differentiation among starch sausage, chicken bone paste, and mixed samples with different ratios. This study provides an effective and rapid method for using LIBS technology to detect elemental composition in foods and distinguish different food samples, offering safety assurance for food production and regulation.

## Figures and Tables

**Table 1:** Possible elemental spectral lines.

Possible Elements	Wavelength (nm)
Ca I	422.67, 430.25, 443.57, 445.48, 458.14, 458.59, 534.95, 558.88, 585.75, 610.27, 612.22, 616.22, 643.91, 646.26, 649.38, 671.77, 714.82, 720.22, 732.61
Ca Ⅱ	315.89, 317.93, 370.60, 373.69, 393.37, 396.85, 849.80, 854.21, 866.21
K I	766.49, 769.90
Mg II	279.55, 280.27
Na I	568.26, 568.82, 589.00, 589.59
Ba II	455.40, 493.41
Sr II	407.77
N I	742.36, 744.32, 746.83, 818.49, 821.63, 856.77, 859.40, 862.92, 868.34, 939.28
O I	777.19, 794.76, 844.64, 926.60
H_α_	656.28
H_β_	486.13
CN	358.40, 358.64, 359.05, 385.10, 385.48, 386.18, 387.18, 388.33, 415.79, 416.73, 418.09, 419.67, 421.59
C_2_	471.45, 473.65, 516.49

**Table 2:** Coefficients of determination for linear fitting under different analytical methods.

Elements	The First Laser Pulse Non-Normalized	The First Laser Pulse Normalized	The Second Laser Pulse Normalized	Correction with Electron Temperature
Ca I	0.302	0.896	0.957	0.972
Ca Ⅱ	0.694	0.877	0.958	0.952
Ba II	0.857	0.818	0.945	0.97
Sr II	0.691	0.842	0.940	0.982

## Data Availability

The original contributions presented in the study are included in the article, further inquiries can be directed to the corresponding author.

## Abbreviations

The following abbreviations are used in this manuscript:

BPNN	Backpropagation neural network
FWHM	Full width at half maximum
ICA	Independent component analysis
LAMP	Loop-mediated isothermal amplification
LIBS	Laser-induced breakdown spectroscopy
LoD	Limit of detection
LSA	Local semantic analysis
NIST	National Institute of Standards and Technology
PCA	Principal component analysis
PCR	Polymerase chain reaction
PLS	Partial least squares
RSD	Relative standard deviation
t-SNE	t-distributed stochastic neighbor embedding

## Appendix A

**Table A1:** The electron temperature corresponding to different samples.

Sample	Electron Temperature (K)
Starch sausage	9655 ± 745
1:9	9984 ± 1809
2:8	9863 ± 913
3:7	9961 ± 954
4:6	8759 ± 772
5:5	9402 ± 890
Chicken bone paste	9583 ± 1613
